# Supplementary material for: Novel embryological classifications of hepatic arteries based on the relationship between aberrant right hepatic arteries and the middle hepatic artery: A retrospective study of contrast-enhanced computed tomography images
Source: PLoS One. 2024 Feb 28;19(2):e0299263. doi: 10.1371/journal.pone.0299263 (PMC10901311; doi:10.1371/journal.pone.0299263)
Supplement: Attachment — Submitted filename: Response to Reviewers.docx [file pone.0299263.s001.docx]

Response to Reviewers

We sincerely appreciate the time and effort you have dedicated to reviewing our manuscript. Your insightful comments and suggestions have been invaluable in enhancing the quality of our work. We are truly grateful for your constructive feedback.

<To Reviewer #1>

1. We added a statement emphasizing that **there is currently no published paper demonstrating the relationship between variant hepatic arteries and MHA** to underscore the significance of our research findings.
2. To validate that the MHA did not branch from the variant LHA, we also needed to prove that i**t did not branch from the replaced LHA**. Therefore, we categorized the cases into two groups: those with replaced LHA and those without.
3. As mentioned in the “Discussion” section, we considered **Type 4 as an overall variation of the hepatic artery rather than an aRHA.** We have now added a clarification that Types 1-3 are defined as aRHA.
4. While our study may lack impact as it primarily derives its findings from **embryological perspectives suggesting that the MHA does not branch from the aRHA,** we believe it serves as an enlightening reminder. Surgeons should always be cautious about the detailed course of variant hepatic arteries during surgery.
5. I have removed the section regarding the clamp test.

<To Reviewer #2>

1. (For the comments 1+3) While our study may lack impact as it primarily derives its findings from e**mbryological perspectives suggesting that the MHA does not branch from the aRHA**, we believe it serves as an enlightening reminder. Surgeons should always be cautious about the detailed course of variant hepatic arteries during surgery.
2. The frequency of RHA for Types 1-4 has been widely reported in the past, and in this study, t**he frequency of each variant did not significantly differ from previous reports** (i..e. Referrence 2,7,8).
3. I have removed the section regarding the clamp test.
