# Supplementary material for: Novel embryological classifications of hepatic arteries based on the relationship between aberrant right hepatic arteries and the middle hepatic artery: A retrospective study of contrast-enhanced computed tomography images
Source: PLoS One. 2024 Feb 28;19(2):e0299263. doi: 10.1371/journal.pone.0299263 (PMC10901311; doi:10.1371/journal.pone.0299263)
Supplement: Attachment — Submitted filename: Response to Reviewers.docx [file pone.0299263.s002.docx]

Response to Reviewers

We sincerely appreciate the time and effort you have dedicated to reviewing our manuscript. Your insightful comments and suggestions have been invaluable in enhancing the quality of our work. We are truly grateful for your constructive feedback.

-There was an error in the values presented in Table 2. The number and percentage of patients with and without rLHA in Type 1 anomaly were mistakenly reversed. (Prior to correction: Type 1 cases with rLHA, MHA arose from CHA in 17 patients<2.2%>; without rLHA, MHA arose from LHA in four patients <0.52%>.) This has been rectified. We apologize for any confusion.

<To Reviewer #4>

1. In this paper, the aberrant RHA (aRHA) is defined as Type 1-3 since the term “aberrant artery” represents the artery with substantially atypical origin or anatomic course. Therefore, Type 4 (Hepatomesenteric trunk) was excluded from aRHAs as it was considered an anomaly of the visceral arterial arches during embryological development. Hepatic arteries in Type 4 are presumed to exhibit the typical branching pattern of ordinary hepatic arteries, MHA is thought to be capable of branching from either RHA or LHA. (We marked up the relevant section in blue, pages 18-19.)
2. We have corrected all the abbreviations, such as HMT, eLHA, eMHA, and eRHA.
3. Similarly, I have also revised those abbreviations within the figures and tables.

Finally, we sincerely thank you again for your insightful feedback.
